# IF THEY BUILD IT, THEY WILL COME: ENGAGING OLDER AFRICAN AMERICANS IN EDUCATION AND RESEARCH

**DOI:** 10.1093/geroni/igac059.867

**Published:** 2022-12-20

**Authors:** Karen Lincoln

**Affiliations:** University of Southern California, Los Angeles, California, United States

## Abstract

Engaging African Americans in health education and research is a mechanism for addressing health disparities and achieving health equity. The science of engagement and retention requires strong community partnerships, authentic leadership, and educational opportunities for students and the broader community. In this presentation, Karen Lincoln will discuss how findings from her research on health and mental health disparities laid the foundation for her community-based health education program and public scholarship. She will highlight Advocates for African American Elders (AAAE), the health education program that she founded and directs. AAAE provides culturally competent health education for older African Americans throughout Los Angeles County and has become a hub for her research, training and mentoring.

